# Assessment of TB47 as a potential novel therapeutic agent: *in vitro* and *in vivo* efficacy against *Mycobacterium leprae*

**DOI:** 10.1128/aac.00318-25

**Published:** 2025-07-23

**Authors:** Gabriel Henrique Fioroni Furlan, Diego Augusto Souza Oliveira, Daniele Ferreira De Faria Bertoluci, Tiago Araujo Gomes, Jonatas Perico, Bruna Leticia Martins, Dejair Caetano Do Nascimento, Suzana Madeira Diorio, Cleverson Teixeira Soares, Shuai Wang, Tianyu Zhang, Patricia Sammarco Rosa, Flavio Alves Lara, Ana Carla Pereira Latini

**Affiliations:** 1 São Paulo State University (UNESP), Medical School 28108 , São Paulo, Brazil; 2 Lauro de Souza Lima Institute 368269, Bauru, São Paulo, Brazil; 3 Oswaldo Cruz Institute, Fiocruz 37903 , Rio de Janeiro, State of Rio de Janeiro, Brazil; 4 Institute of Drug Discovery, State Key Laboratory of Respiratory Disease, Guangzhou Institutes of Biomedicine and Health, Chinese Academy of Sciences 74627, Guangzhou, China; 5 Guangdong-Hong Kong-Macao Joint Laboratory of Respiratory Infectious Diseases, Guangzhou Institutes of Biomedicine and Health, Chinese Academy of Sciences 74627, Guangzhou, China; 6 China-New Zealand Joint Laboratory on Biomedicine and Health, Guangzhou Institutes of Biomedicine and Health, Chinese Academy of Sciences 74627, Guangzhou, China; 7 University of the Chinese Academy of Sciences 74519, Beijing, China; 8 Guangzhou National Laboratory 612039, Guangzhou, China; Bill & Melinda Gates Medical Research Institute, Cambridge, Massachusetts, USA

**Keywords:** leprosy treatment, mycobacterial ETC inhibitors, multidrug therapy, synergistic effect

## Abstract

Leprosy is a chronic infectious disease caused by *Mycobacterium leprae* and *Mycobacterium lepromatosis*. The treatment typically involves multidrug therapy comprising dapsone, clofazimine, and rifampicin for 6–12 months. TB47 is a new inhibitor of the mycobacterial electron transport chain (ETC), disrupting ATP production in bacteria. This study investigated the *in vitro* and *in vivo* antimicrobial effects of TB47 on *M. leprae. In vitro* assays employed IDE8 tick cells infected with *M. leprae*, showing that after 30 days of infection, 5 ng/mL of TB47 treatment significantly impaired bacillary growth. For *in vivo* assays, BALB/c mice were infected with *M. leprae* and subjected to different treatments with varying doses of TB47 combined or not with clofazimine. Treatments were administered weekly for 90 days (13 times). The effects were assessed immediately after treatment, as well as at 120 and 210 days post-treatment. Results showed that 100 and 10 mg/kg of TB47 combined with 5 mg/kg clofazimine exhibited a bactericidal effect on *M. leprae* in all time points evaluated, in contrast with clofazimine monotherapy, for which the bactericidal effect was observed only 210 days post-treatment. TB47 monotherapy had a bacteriostatic effect immediately after treatment, but replication resumed at later evaluation points. Histopathological evaluation supported these findings. Combining dose-dependent TB47 with clofazimine showed an additive bacteriostatic and bactericidal effect on *M. leprae*, suggesting an advantageous pharmacokinetic and pharmacodynamic profile. Further research into mycobacterial ETC inhibitors could significantly impact leprosy management by providing more effective and shorter treatment options.

## INTRODUCTION

Leprosy is a chronic infectious disease that affects the peripheral nerves and the skin, caused by *Mycobacterium leprae (M. leprae*) or *Mycobacterium lepromatosis*. In 2023, the World Health Organization (WHO) reported 182,815 new cases of leprosy worldwide. Notably, 79.3% cases were reported in India, Brazil, and Indonesia. This report also revealed 13,777 retreatment cases, indicating the urgency for the development of new and effective drugs to control the disease (1).

Leprosy is classified according to the bacillary burden, clinical aspects of lesions, and histological findings, which are largely influenced by the host immune response (2). The regular leprosy treatment is a multidrug therapy (MDT) composed of dapsone (DDS), clofazimine (CFZ), and rifampicin (RFP) for 6 months to 1 year. The lengthy treatment regimen and the bacillary resistance to the MDT drugs are the main issues associated with retreatment and relapse rates. In cases of proven drug resistance, alternative therapies including minocycline, ofloxacin, levofloxacin, or clarithromycin are prescribed (3).

A new class of drugs have been explored to address leprosy treatment issues, expanding the arsenal of antimycobacterial agents. Antimycobacterial drugs targeting oxidative phosphorylation (OXPHOS) are of interest due to their ability to deprive the central metabolism of the bacilli (4). These drugs were initially tested in tuberculosis treatment with bedaquiline, which inhibits the F-ATP synthase complex, thereby inhibiting ATP production. Bedaquiline is the first new anti-tuberculosis drug approved in more than 50 years, and its bactericidal activity against *M. leprae* has already been demonstrated (5–7). Q203 (known as Telacebec) is an electron transporter chain (ETC) inhibitor with activity against mycobacteria, including *M. leprae* (8, 9). It binds within the cytochrome bc1-aa3 supercomplex and promotes a decline in intracellular ATP synthesis. Both *in vitro* and *in vivo* experiments have shown that *M. leprae* is sensitive to the Q203 molecule (8).

Pyrazolo[1,5 a]pyridine-3-carboxamide, abbreviated as TB47, is a structural analog of Q203 with the same mechanism of action. Both compounds bind the site responsible for the oxidation of menaquinol to menaquinone in the cytochrome bc1 complex’s cytochrome b subunit (QcrB) within the cytochrome bc1-aa3 supercomplex (10). Menaquinone is essential for shuttling electrons through the ETC to produce the electrochemical gradient required for ATP production (11).

The utilization of the alternative terminal cytochrome oxidase bd by bacteria diminishes the efficacy of QcrB inhibitors. However, *M. leprae* has lost the genes encoding the cytochrome bd oxidase and other alternative terminal electron acceptors, potentially rendering it reliant on the cytochrome bc1-aa3 terminal oxidase for respiration (12, 13). Thus, QcrB inhibitors, such as Q203 and TB47, emerge as promising agents in the treatment of leprosy.

Although the effect of TB47 and clofazimine combination against *Mycobacterium abscessus* is controversial (14, 15), clofazimine exhibits a synergistic effect with TB47 in *Mycobacterium tuberculosis* models (16, 17), likely due to its ability to compete with menaquinone by shuttling electrons from type II NADH dehydrogenase (NDH-2) to oxygen, thereby generating lethal levels of reactive oxygen species (ROS) in microorganisms (18). This mechanism underscores the potential of combining clofazimine with cytochrome bc1 inhibitors to enhance the elimination of both replicating and nonreplicating *M. tuberculosis* (19), which is crucial for treating latent infections. Although the concept of latency in leprosy remains elusive, it may contribute to the high relapse rates observed in patients (1). In our study, we evaluated the *in vitro* antimicrobial activity of TB47 against *M. leprae* using an established maintenance model (20) and conducted *in vivo* assessments via the Sheppard model (21). Our findings indicate that TB47, when used in conjunction with clofazimine, exerts an additive bactericidal action against *M. leprae*.

## MATERIALS AND METHODS

### Antimicrobials

The TB47 compound was synthesized and supplied by Guangzhou Eggbio Co., Ltd. with a purity of 98.67% (Batch number: TB47160616). Clofazimine (CFZ) was obtained from Sigma-Aldrich (CAS-2030-63-9) (San Louis, Missouri, EUA), and carboxymethyl cellulose (CMC) was obtained from Nutrifarm (São Paulo, Brazil).

### *M. leprae* strains

The *M. leprae* Thai 53 strain was obtained from the footpads of athymic mice (athymic, nu/nu, Foxn1nu). The strain is maintained in the animal facility of the Lauro de Souza Lima Institute. The footpads were excised and macerated in saline using an UltraTurrax tissue homogenizer (IKA, North Carolina, United States). Bacillary counts were performed on microscope slides stained with cold Ziehl-Neelsen, as described by Trombone et al. (22). The concentration of bacilli was adjusted to inoculate 1 × 10^4^ bacilli in 30 µL into both hind footpads of each BALB/c mouse.

### *In vitro* assay

The *in vitro* model employed IDE8 cells from *Ixodes scapularis* ticks (20). The cells were obtained through collaboration with Dr. Lesley Bell-Sakyi from the Tick Cell Biobank of the Institute of Infection and Global Health at the University of Liverpool (Liverpool, United Kingdom). Cells were cultivated in a Leibovitz’s L-15 modified medium, L15B (23) in 24-well plates at 2 × 10^5^ cells/well and infected with 10^7^ *M. leprae* obtained from footpads of athymic mice, resulting in a multiplicity of infection (MOI) of 50 bacilli for each cell. After 48 h of infection, TB47 or rifampicin was added to the medium using 1% dimethyl sulfoxide (DMSO) as the vehicle. Infected untreated cells and cells exposed to 1% DMSO were also evaluated as controls. Cultures were maintained at 30°C, with medium changes containing either drugs or vehicle performed every 7 days. After 30 days of culturing (representing 28 days of infection), the cells were lysed using 0.1% Triton X-100 (Sigma Aldrich, St. Louis, Missouri, USA). The bacilli enumeration was performed according to the Sheppard & McRae (1968) method (24). Briefly, cell extracts were pelleted by centrifugation at 14,000 × *g* for 5 min at room temperature and resuspended in 50 µL of formaldehyde-milk. Bacillary suspensions were evaluated by optical microscopy after dissociation, achieved by passing the preparation through a 26G needle 10 times, followed by dilution at 1:2, 1:10, and 1:100 in formaldehyde-milk.

Cold Ziehl-Neelsen staining was performed according to the manufacturer’s instructions (BD, Franklin Lakes, New Jersey, USA) after drying 10 µL of each dilution onto slides containing 10 mm etched rings (EMS, Hatfield, Pennsylvania, USA). To quantify *M. leprae* titers, we used a 100 × Plan Apo objective attached to a Z1 AxioObserver microscope (Zeiss, Oberkochen, Germany).

The median of the count of ten fields was used for quantification. In our setup, the lower limit of detection, defined as one bacillus observed across ten fields at the 1:2 dilution, was determined to be 4,380 bacilli.

### *In vivo* assay

For the *in vivo* assay, 140 8-week-old female BALB/c mice were infected with 10⁴ *M. leprae* in both hind footpads. Sixty days post-inoculation, five animals were euthanized, revealing a bacillary load of 8.6 × 10⁴ (± 5.3 × 10⁴) bacilli per footpad.

After confirmation of infection, the animals were divided into eight groups, one untreated control group and seven treatment groups: CFZ 5 mg/kg; TB47 100 mg/kg combined with CFZ 5 mg/kg; TB47 10 mg/kg combined with CFZ 5 mg/kg; TB47 1 mg/kg combined with CFZ 5 mg/kg; TB47 100 mg/kg; TB47 10 mg/kg and TB47 1 mg/kg.

The standard kinetic method described by Sheppard (25) was used to evaluate the bacteriostatic or bactericidal effect of the compounds. The animals were treated once a week for 90 days (13 times). The treatments were performed during the logarithmic multiplication phase of the bacilli, from days 60 to 150 after infection. Mice were euthanized at the end of treatment or 150 days after infection (T1), 120 days after the end of treatment or 270 days after infection (T2), and 210 days after the end of treatment or 360 days after infection (T3). This method considers bacillary replication positive when more than 10^5^ bacilli per footpad are recovered. A bacteriostatic effect occurs when no bacterial growth is observed only during the time the drug is being administered, but growth resumes once the treatment ends. In contrast, a drug is considered bactericidal if bacterial growth does not resume even after the treatment has stopped (21).

All drugs were dissolved in 0.05% CMC solution and administered by gavage in a volume of 0.2 mL, except for CFZ 5 mg, which was administered in a volume of 0.1 mL.

The left footpads were placed in 1 mL of 10% buffered formalin for histopathological analysis by hematoxylin-eosin and Fite-Faraco staining. The right footpads were processed following the method outlined by Trombone et al. (22). From the total volume of 2 mL, 50 µL was used for bacillary enumeration after performing cold Ziehl-Neelsen staining, as described above. The remaining sample was centrifuged at 14,000 *g* and 4°C for 30 minutes. The resulting pellets were then stored in 500 µL of QIAzol (Qiagen, Hilden, Germany) at −80°C until nucleic acid extractions were conducted.

### Nucleic acid extraction

Nucleic acids were extracted using a protocol adapted from Collins et al. (26). The pellets obtained from footpads processing were thawed and homogenized in FastPrep-24 equipment using Lysing Matrix D tubes (MP Biomedicals, Santa Ana, CA, USA), in two cycles of 6,5 m/s for 45 seconds (s) with a 5 min interval on ice. Next, 400 µL of chloroform: isoamyl alcohol 24:1 (Merck, Darmstadt, Hesse, Germany) was added to the microtubes and left at room temperature for 3 min and then centrifuged at 700 g for 5 min at 4°C. All the contents were transferred to new microtubes and centrifuged at 14,000 *g* for 10 min at 4°C. For the RNA isolation, 400 µL of the superior aqueous phase was transferred to a new microtube, and 400 µL of chloroform: isoamyl alcohol 24:1 was added. The mixture was vortexed for 5 s and left at room temperature for 3 min. The samples were centrifuged at 14,000 *g* for 2 min at 4°C, and the aqueous phase was transferred to new microtubes. Then, 2 µL of glycogen (Invitrogen, Waltham, Massachusetts, USA), 30 µL of 5 M ammonium acetate (Merck, Darmstadt, Hesse, Germany), and twice the collected volume of cold isopropanol (Merck, Darmstadt, Hesse, Germany) were added. The mixtures were incubated overnight at −80°C. After that, the samples were centrifuged at 14,000 *g* for 30 min at 4°C and the supernatants discarded. Two washes were performed with 1 mL of ice-cold 75% ethanol (Merck, Darmstadt, Germany) and centrifugation at 12,000 *g* for 5 min at 4°C. The microtubes were inverted to dry, and the pellets were resuspended in 25 µL of nuclease-free water. The RNA was treated twice with TURBO DNase (INVITROGEN, Carlsbad, CA, USA) by adding 2 µL of DNase and 3 µL of the buffer provided with the kit. The samples were incubated at 37°C for 30 min, and the process was repeated. Finally, the samples were incubated at 70°C for 10 min for enzyme inactivation.

The protocol for the DNA precipitation was based on Collins et al. (26). Briefly, Tris-EDTA buffer and chloroform: isoamyl alcohol 24:1 (Merck, Darmstadt, Hesse, Germany) were added to the tubes from which RNA was isolated from the superior phase and subjected to FastPrep-24 (MP-Biomedicals, Irvine, California, USA). The aqueous phase was transferred to a new microtube, and DNA precipitation was performed by adding glycogen (Invitrogen, Massachusetts, USA), 5 M ammonium acetate (Merck, Darmstadt, Hesse, Germany), and cold absolute ethanol (Merck, Darmstadt, Hesse, Germany). The mixtures were incubated overnight at −80°C and centrifuged, and the pellets were washed with 70% ethanol (Merck, Darmstadt, Hesse, Germany) and resuspended in 30 µL of Tris-EDTA buffer.

### Molecular determination of *M. leprae* viability

Molecular viability assays were performed following the protocol described by Collins et al. (26), based on *M. leprae hsp18* and *esxA* gene expression. These genes encode the 18 kD heat shock protein (hsp18) and ESAT-6 proteins, respectively. This protocol involves adjusting the quantity of RNA to transcription according to the amount of bacillary DNA in the sample.

Briefly, we performed quantitative PCR (qPCR) using Roche SYBR Green Master Mix (Merck, Darmstadt, Germany) on a CFX Opus 96 system (Bio-Rad, Hercules, CA, USA), following the manufacturer’s protocols. Initial bacillary DNA quantification used an 8-point standard curve (1:4 serial dilutions) of pUCIDT-RLEP plasmid. RNA equivalent to 3,000 bacilli was calculated and transcribed using M-MLV Reverse Transcriptase (Sigma-Aldrich, St. Louis, MO, USA) in a PTC-100 thermal cycler (Marshall Scientific, Hampton, VA, USA). Molecular viability was assessed through hsp18 and esxA expressions using 8-point standard curves from two plasmids (containing hsp18/esxA or 16S sequences), with 16S rRNA serving as the RNA quality control (26). All plasmids were generously provided by Dr. Linda B. Adams (National Hansen’s Disease Program, Louisiana, USA).

### Data analysis

Data were evaluated for normality using the Shapiro-Wilk test. For inter-group comparisons, parametric data were analyzed with Student’s *t*-test, while nonparametric data were analyzed with the Mann-Whitney U test. When comparing three or more groups, parametric data were assessed through ANOVA, followed by Tukey’s *post hoc* test, and nonparametric data were examined using the Kruskal-Wallis test supplemented with Dunn’s *post hoc* analysis. All statistical analyses were conducted employing GraphPad Prism 9 software, adopting a significance threshold of *P* < 0.05.

## RESULTS

**Fig 1:**
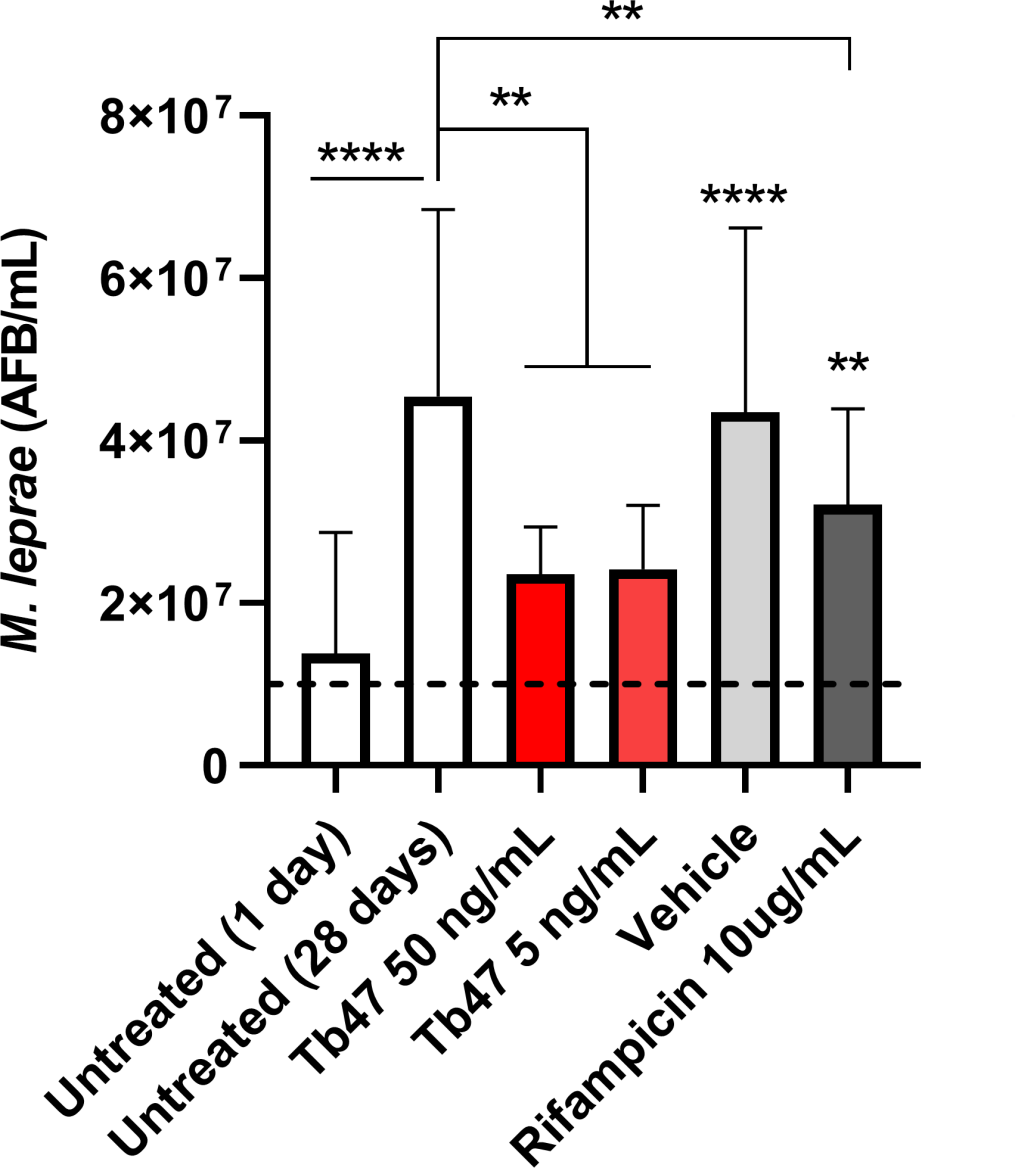
TB47 inhibits *M. leprae* growth in the IDE8 tick cell line. The IDE8 tick cell line was infected with *M. leprae* (10^7^ bacilli, dashed line) under six different conditions: untreated controls, vehicle (DMSO 1%), TB47 at 50 ng/mL, TB47 at 5 ng/mL, and rifampicin at 10 µg/mL. The cultures were maintained with medium changes, containing the drugs or vehicle, every 7 days. After 30 days, the bacillary load was assessed by Ziehl-Neelsen staining. Each bar represents the results from three independent experiments. Statistical analysis was performed using unpaired one-way ANOVA and Kruskal-Wallis multiple comparisons to the 28 day untreated group, unless otherwise indicated, *P* < 0.001 (**) and *P* < 0.0001 (****). Unless otherwise indicated, asterisks represent statistically significant differences from the untreated (1 day) control group.

For rational use of animals, we initially conducted an *in vitro* assay to evaluate TB47’s potential against *M. leprae*. The ability of *M. leprae* to multiply in a cell line derived from *Ixodes scapularis* tick (IDE8) is already well-established (20). In the present study, we observed *M. leprae* growth from approximately 10⁷ to slightly more than 4 × 10⁷ over 28 days. On the other hand, IDE8 cells treated during this period with rifampicin 10 ug/mL or TB47 at 5 or 50 ng/mL significantly impaired bacillary growth compared to untreated infected cells (Fig. 1).

**Fig 2:**
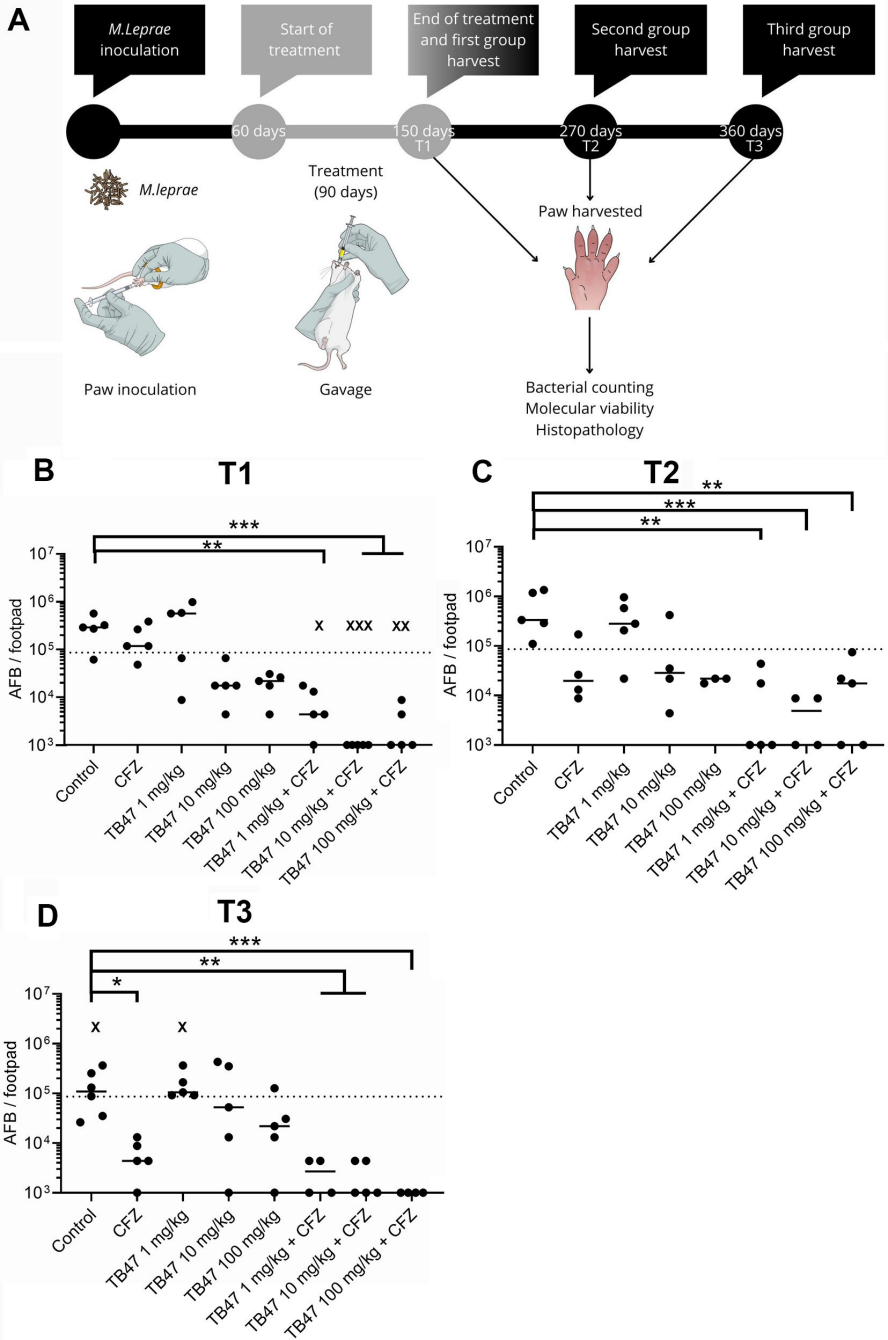
*In vivo* experimental timeline and treatment efficacy evaluation. (**A**) BALB/c mice were infected with *M. leprae* (10⁴ bacilli, dashed line) in each hind footpad and divided into seven different treatment regimens along with an untreated control group. Treatment was administered from day 60 to day 150 post-infection. Mice were euthanized at different time points: at the end of treatment (150 days post-infection, T1), 120 days after treatment completion (270 days post-infection, T2), and 210 days after treatment completion (360 days post-infection, T3). (B–D) The number of acid-fast bacilli (AFB) recovered from mouse footpads was counted using Ziehl-Neelsen staining. Data are presented as mean ± standard deviation at the following time points: T1 (**B**), T2 (**C**), and T3 (**D**). CFZ refers to clofazimine 5 mg/kg. Statistical analysis was performed using unpaired one-way ANOVA and Kruskal-Wallis multiple comparisons, where *P* < 0.005 (*), *P <* 0.001 (**), and *P* < 0.0005 (***). Unless otherwise indicated, asterisks represent statistically significant differences from the clofazimine group. Groups containing fewer than five animals indicate mortality occurring during the study period.

Next, we extended these findings to infected BALB/c mice using the Sheppard model, comparing treatments to the control group (Fig. 2A). Immediately after treatment/150 days post-infection (T1), we found that all combinations of TB47 with clofazimine (5 mg/kg) prevented bacillary replication, with 9 of 15 animals showing virtually no *M. leprae*, which means loads below 4 × 10³ bacteria per footpad. These observations demonstrate an additive effect of TB47 compared to clofazimine alone at this time point, which failed to control *M. leprae* replication when administered as monotherapy at this suboptimal concentration (Fig. 2B). At this time point, TB47 monotherapy in all dosages also failed to control *M. leprae*.

To determine whether the observed effect was mycobactericidal or mycobacteriostatic, we assessed bacterial replication at 270 days post-infection (T2; 120 days post-treatment). While we observed low *M. leprae* titers in all groups treated with TB47-clofazimine combinations (Fig. 2C), only half of these animals showed undetectable *M. leprae* loads in footpads. At both time points, no significant differences were found between untreated controls and groups receiving either clofazimine (5 mg/kg) or TB47 alone.

The final evaluation at 360 days post-infection (T3, 210 days post-treatment) confirmed the bactericidal effect of TB47 combined with clofazimine (5 mg/kg), with 9 of 13 animals showing undetectable levels of *M. leprae* (Fig. 2D). At this time point, clofazimine monotherapy (5 mg/kg) also showed statistically significant differences from the control group, but with only 1 of 5 animals demonstrating the virtual absence of *M. leprae*.

**Fig 3:**
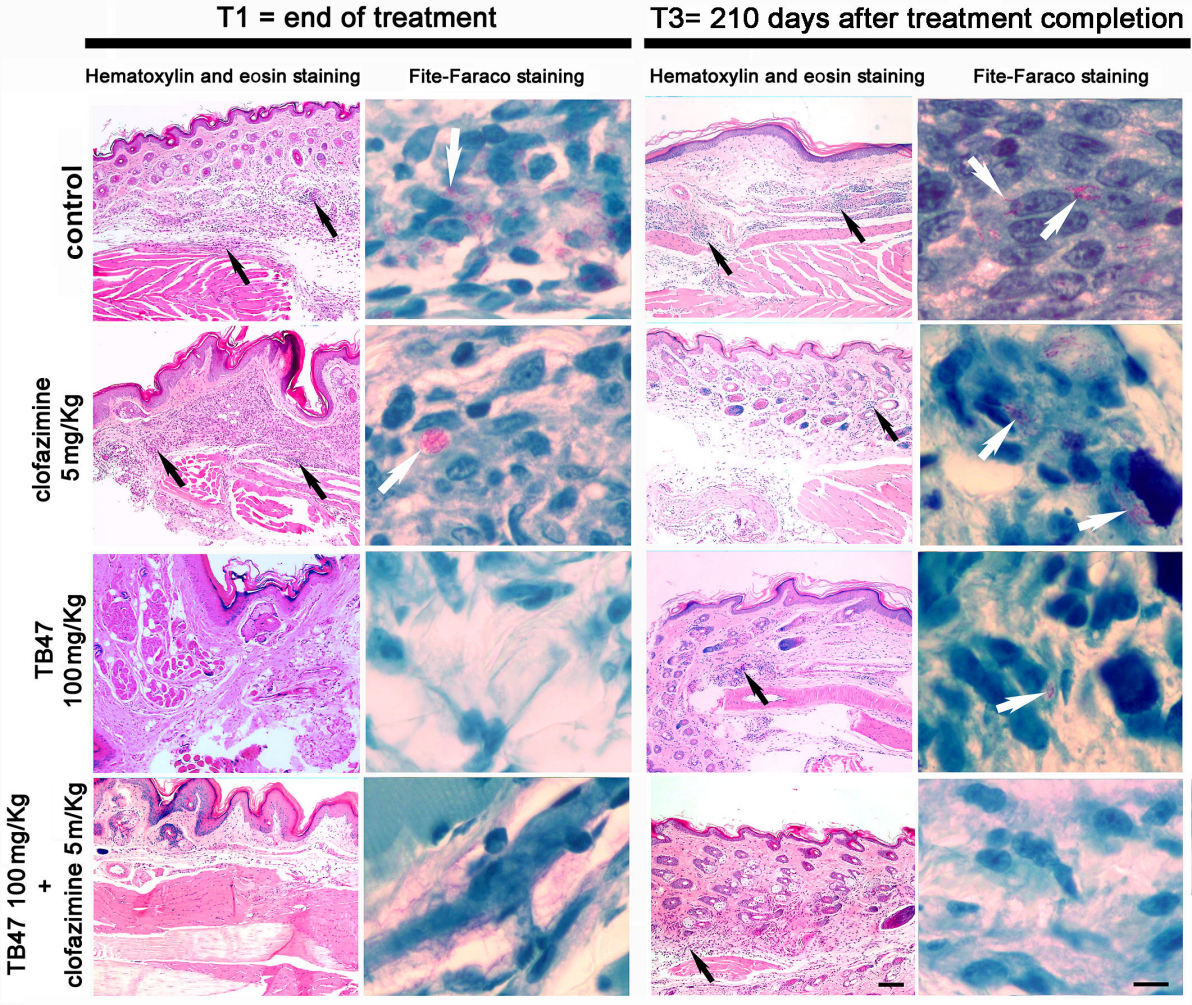
Dermis inflammatory chronic granulomatous lympho-histiocytic process and acid-fast bacilli persistence along the different treatments. Dermis of BALB/c mice footpads after 90 day treatment (T1) or 210 days after the end of the treatment (T3) were stained by hematoxylin and eosin to demonstrate inflammatory infiltration (black arrows) involving vessels and skeletal muscles or Fite-Faraco staining to evidence acid-fast bacilli (white arrows). The left scale bar represents 100 µm, and the right scale bar represents 10 µm.

The molecular viability analysis, determined by *M. leprae hsp18* and *exsA* transcript levels, proved unsuitable for the Sheppard model employed in our study (21). While we anticipated that the 60-day incubation period prior to treatment would be sufficient to yield detectable *M. leprae hsp18* and *exsA* transcription, more than 90% of the samples, including untreated controls, presented signal below the assay’s sensitivity threshold (see Table S1 at https://doi.org/10.5281/zenodo.15652029). The histological pattern in mice inoculated with *M. leprae* did not vary greatly between the groups and times. Most of them showed diffuse lympho-histiocytic granulomatous infiltrates penetrating the dermis and skeletal muscle layer. Nerves could be observed in most examined slides. The differences observed were related to the size of the inflammatory infiltrate on hematoxylin and eosin staining and the bacillary index (BI) on Fite-Faraco-stained slides. (Fig. 3 and see Table S1 at https://doi.org/10.5281/zenodo.15652029).

In the control group, inflammatory infiltrates were more pronounced compared to the other groups, often showing the presence of neutrophils. The bacillary index (BI) ranged from 6 + at T1, with well-stained bacilli, to 2+/4 + at T3, with a greater number of fragmented bacilli. Monotherapy with clofazimine 5 mg/kg exhibited a high bacillary burden at T1, which decreased slightly at T3. The monotherapies using 100 mg/kg of TB47 showed mild-to-moderate infiltration in both time points. The BI for the 100 varied from negative at T1 to 4 + at T3. The group treated with 1 mg/kg of TB47 consistently displayed a minimum BI of 4 + with well-stained bacilli at every time point and moderate to intense inflammatory infiltrates (data not shown).

Notably, the group treated with the combination therapy containing TB47 100 mg/kg with clofazimine 5 mg/kg was the only one to record a negative BI at all time points. Conversely, the group receiving the combination of clofazimine 5 mg/kg with TB47 10 mg/kg showed a BI 2 + of fragmented bacilli at T2, also achieving a negative BI at T3 (data not shown). The group treated with the combination clofazimine 5 mg/kg and TB47 1 mg/kg showed a positive BI at T3, with levels reaching 4 + to 5 + alongside moderate inflammatory infiltrates (data not shown).

## DISCUSSION

In the present study, we conducted an *in vitro* analysis to guide a larger *in vivo* study investigating the anti-*M. leprae* activity of a pyrazolo[1,5 a]pyridine-3-carboxamide compound named TB47. By quantifying the number of *M. leprae* within the IDE8 cell line derived from the *Ixodes scapularis* tick (20), we assessed the drug’s capacity to kill or inhibit bacillus growth.

Unfortunately, this low-cost *in vitro* technique has intrinsic limitations related to *M. leprae* biology. For example, inactivated *M. leprae* takes several days to fragment and to become non-stainable. Consequently, the number of stainable *M. leprae* can still be observed at the end of the experiment, even after 30 days of exposure to rifampicin. For the same reason, it is challenging to observe significant differences in *M. leprae* numbers between high and low doses of TB47. However, a significant difference between the control and treated conditions can still be interpreted as evidence of bactericidal or bacteriostatic activity. Despite these challenges, we have successfully relied on this straightforward test as a preliminary step in the search for new anti-*M. leprae* drugs before initiating lengthy studies involving animals and large quantities of drugs.

New antimycobacterial drugs targeting the mycobacterial OXPHOS show promise in reducing treatment durations for diseases such as Buruli ulcer, leprosy, and tuberculosis (27). Studies evaluating the cytochrome bc1-aa3 supercomplex inhibitors TB47 and Q203, in combination with some antimycobacterial agents, have shown promising results for this purpose, with strong synergistic effects with clofazimine, a weaker synergistic effect with ethambutol, and no significant potentiation of amikacin, levofloxacin, or linezolid bactericidal effects (14–17).

We demonstrated for the first time that the clofazimine-QcrB inhibitor combination has a bactericidal effect against *M. leprae*. Our results support an additive effect model for combining TB47 with clofazimine. A sterilizing effect of TB47 and clofazimine has also been described in a murine model of infection for *M. tuberculosis* (16, 17) and for *M. ulcerans* (27, 28). This result is in agreement with other data indicating that although alone, TB47 presented no mycobactericidal effect, and in association with clofazimine, TB47 can shorten the treatment duration in comparison to rifampicin alone (10).

Unfortunately, qPCR-based viability determination has an estimated sensitivity of 1.785 × 10³ for hsp18 and 5.395 × 10³ for esxA in mouse footpads (29). Given that Sheppard’s model involves inoculating 10⁴ bacilli into the footpads of immunocompetent BALB/c mice (30), most of the analyzed samples yielded results near or below the sensitivity cutoff, limiting our viability analysis.

*M. leprae* enumeration along treatment indicates that TB47, when applied once weekly, exhibits a dose-dependent bacteriostatic effect on *M. leprae*. This bacteriostatic effect of TB47 monotherapy was also observed in a murine model of *M. tuberculosis* infection (10, 17). *M. leprae* lacks genes encoding the cytochrome bd oxidase, and if it relies solely on the cytochrome bc1-aa3 terminal oxidase for respiration, the cytochrome bc1-aa3 terminal oxidase inhibitors, such as TB47 and Q203, should be bactericidal against *M. leprae,* just as observed in the case of *M. ulcerans* (29, 31, 32).

Data regarding the *in vivo* effect of Q203 against *M. leprae* indicate that monotherapy led to the death of 99% of bacilli after administering 20 doses, which was not achieved with five doses. The Lahiri et al. (8) study was based only on the molecular viability immediately after treatment and did not assess bacilli recovery. They conclude that monotherapy with Q203 does not exert a bactericidal effect against *M. leprae* when administered in limited doses (8).

For that reason, it is reasonable to anticipate that TB47 will exhibit only bacteriostatic activity against *M. leprae* in our mouse model. There may be two possible explanations for these observations. One is that an alternative terminal oxidase, which is different from both the cytochrome bd and bc1-aa3 terminal oxidases, may exist in *M. leprae,* and its activity can be influenced by clofazimine. The other is that here we only used TB47 weekly, and so the effective concentration in the footpads may not be high enough since the half-life of TB47, 35.6 h by oral administration, is not as long as that of CFZ (10). In contrast, rifampicin, a first-line leprosy drug with a short half-life of only 2.5 hours (33), still demonstrates potent bactericidal activity in this animal model when administered weekly (34).

Clofazimine has demonstrated effectiveness against *M. leprae*, even at low doses of 0.25 mg/kg per day, with the bactericidal activity exceeding 84% in experimental models (35). When clofazimine was administered at a continuous dose of 25 mg/kg per day, or an intermittent dose of 75 mg/kg per day, it significantly affected the viability of the bacillus after 100 days of treatment (36).

Notably, a *M. leprae* strain resistant to dapsone and rifampicin remained sensitive to clofazimine when given at a dose of 2.5 mg/kg daily (37). The effects of clofazimine treatment were observed to continue even after the treatment had ended. Since clofazimine accumulates inside macrophages (38) and remains active post-treatment, we can infer that clofazimine has a slower/accumulative bactericidal impact on *M. leprae* physiology.

Probably for this reason, we observed clofazimine failing to eradicate *M. leprae* at T1 and T2, but successfully controlling the infection at T3. This long-lasting effect, probably restricted to the intracellular environment, was also noted in comparison to other drugs (30). These observations may explain why clofazimine alone successfully eliminated the bacilli at the T3 time point, but only when combined with TB47 was it able to eliminate the bacilli at time points T1 and T2.

At T1 and T2 time points, weekly doses of TB47 combined with clofazimine significantly reduced the bacillary load compared to untreated controls or TB47 or clofazimine monotherapies. These data suggest that the mycobactericidal effect of the TB47-clofazimine combination is TB47 dose-dependent and, therefore, not solely attributable to clofazimine.

A key limitation of our study stems from the inability to culture *M. leprae* in cell-free systems. Consequently, we assessed the drug efficacy by combining bacillary enumeration with histopathological analysis of footpad tissues. While we employed molecular viability testing based on *M. leprae hsp18* and *exsA* transcript levels, this method requires a DNA input equivalent to 1.9 × 10⁴ bacilli (29), a threshold not reached in most BALB/c footpad samples. As a result, molecular viability assessment was not feasible across all samples. Nevertheless, histopathological examination confirmed the absence of *M. leprae* in footpads from animals treated with clofazimine 5 mg/kg and TB47 100 mg/kg.

In conclusion, our study highlights the significant bacteriostatic effect of weekly TB47 monotherapy against *M. leprae* and its bactericidal effect when combined with clofazimine. Even the lowest dose of TB47 tested (1 mg/kg), when combined with clofazimine 5 mg/kg, exhibited a notable bactericidal effect at all time points evaluated. While the limitation of not being able to cultivate *M. leprae* in a cell-free system posed challenges, our data provide robust evidence supporting clinical studies regarding the use of TB47 in leprosy treatment protocols to address bacillary resistance and to reduce therapy duration. Clinical trials to evaluate the efficacy and safety of TB47 for tuberculosis treatment are planned to start in 2025.
